# Chemical Profile of *Launaea nudicaulis* Ethanolic Extract and Its Antidiabetic Effect in Streptozotocin-Induced Rats

**DOI:** 10.3390/molecules26041000

**Published:** 2021-02-13

**Authors:** Samah A. El-Newary, Sherif M. Afifi, Mohamed S. Aly, Rania F. Ahmed, Abd El-Nasser G. El Gendy, Ahmed M. Abd-ElGawad, Mohamed A. Farag, Abdelbaset M. Elgamal, Abdelsamed I. Elshamy

**Affiliations:** 1 Medicinal and Aromatic Plants Research Department, National Research Centre, 33 El Bohouth St., Dokki, Giza 12622, Egypt; samahelnewary@yahoo.com (S.A.E.-N.); aggundy_5@yahoo.com (A.E.-N.G.E.G.); 2 Pharmacognosy Department, Faculty of Pharmacy, University of Sadat City, Sadat City 32897, Egypt; shshsh38@hotmail.com; 3 Department of Animal Reproduction and Artificial Insemination, National Research Centre, 33 El Bohouth St., Dokki, Giza 12622, Egypt; mohamedaly_nrc@yahoo.com; 4 Chemistry of Natural Compounds Department, National Research Centre, 33 El Bohouth St., Dokki, Giza 12622, Egypt; rfawzi@hotmail.com (R.F.A.); elshamynrc@yahoo.com (A.I.E.); 5 Department of Botany, Faculty of Science, Mansoura University, Mansoura 35516, Egypt; 6 Pharmacognosy Department, College of Pharmacy, Cairo University, Kasr el Aini St., Cairo P.B. 11562, Egypt; mfarag73@yahoo.com; 7 Chemistry Department, School of Sciences & Engineering, The American University in Cairo, New Cairo 11835, Egypt; 8 Department of Chemistry of Microbial and Natural Products, 33 El-Bohouth St., Dokki, Giza 12622, Egypt

**Keywords:** *Launaea nudicaulis*, antihyperglycemic, liver and kidney functions, histopathological studies, metabolites profiling, LCMS

## Abstract

*Launaea nudicaulis* is used in folk medicine worldwide to treat several diseases. The present study aimed to assess the antidiabetic activity of *L. nudicaulis* ethanolic extract and its effect on diabetic complications in streptozotocin-induced hyperglycemic rats. The extract was orally administrated at 250 and 500 mg/kg/day for 5-weeks and compared to glibenclamide as a reference drug at a dose of 5 mg/kg/day. Administration of the extract exhibited a potential hypoglycemic effect manifested by a significant depletion of serum blood glucose concurrent with a significant elevation in serum insulin secretion. After 5-weeks, extract at 250 and 500 mg/kg/day decreased blood glucose levels by about 53.8 and 68.1%, respectively, compared to the initial values (*p* ≤ 0.05). The extract at the two dosages prevented weight loss of rats from the 2nd week till the end of the experiment, compared to diabetic control rats. The extract further exhibited marked improvement in diabetic complications including liver, kidney and testis performance, oxidative stress, and relative weight of vital organs, with respect to diabetic control. Histopathological examinations confirmed the previous biochemical analysis, where the extract showed a protective effect on the pancreas, liver, kidney, and testis that degenerated in diabetic control rats. To characterize extract composition, UPLC-ESI–qTOF-MS identified 85 chromatographic peaks belonging to flavonoids, phenolics, acyl glycerols, nitrogenous compounds, and fatty acids, with four novel phenolics reported. The potential anti-diabetic effect warrants its inclusion in further studies and or isolation of the main bioactive agent(s).

## 1. Introduction

Diabetes mellitus (DM) is a long-term condition and one of the degenerative diseases affecting the life-quality of individuals and their families and societies. This disease is one of the most chronic widespread community diseases worldwide. In 2019, the global diabetes prevalence was estimated at 9.30% (463 million people), which could reach 10.20% (578 million people) by 2030 and 10.90% (700 million people) by 2045 [1,2]. Worldwide, Egypt, with 8.9 million people, is considered one of the top five countries in the Middle East and North Africa for diabetes prevalence, according to Thomas et al. [2]. Diabetes mellitus is one of the main reasons for glucose autoxidation, protein glycation, and polyol metabolism activation. These syndromes cause the acceleration of reactive oxygen species generation that increases the levels of oxidized forms of DNA, proteins, and lipids in several body tissues and therefore increases oxidative stress. All the syndromes of DM and its complications can be basically correlated with the oxidative stress [3]. There is a positive relation between STZ-induced hyperglycemia (type-1) and oxidative stress resulting in diabetes complications, including alterations in tissue, lipid peroxidation, protein inhibition, and glycation [4]. Also, there is a relation between insulin resistance and oxidative stress, causing a disruption in glucose and lipid metabolism concurrent with inhibition of the antioxidant enzyme system. Consequently, many antioxidant chemicals exhibit hypoglycemic effects that enable them to be used as diabetes medications. Several chemical hypoglycemic drugs are used, including insulin, sulfonylureas, metformin, Na+-glucose exchanger inhibitors, meglitinides, GLP-1 agonists, dipeptidyl peptidase-4 inhibitors, insulin analogs and *α*-glucosidase inhibitors. These drugs have, nevertheless, side effects warranting the search for new hypoglycemic natural compounds with less side effects [5]. Reproductive dysfunction and male testicular damage are consequences of DM in both humans and animals. DM decreases testicular weight, sperm count, motility, and plasma testosterone levels, along with alterations of the spermatogenesis process [6], induces cellular apoptosis, and triggers male infertility [7]. Nevertheless, the testicular histopathological alterations has never been deeply studied and these examinations could possibly aid in judging the efficacy and safety of newly emerged antidiabetic agents.

Medicinal plants are commonly used in traditional medicine worldwide due to their distribution, safety, efficacy, and relatively low costs [8]. *Launaea nudicaulis* (L.) Hooker fil. is commonly known as Al-Hewa in the Arabic region. Its leaves are used in folk medicine for the treatment of children’s fever, skin itches, eczema, swelling, bilious fever, ulcers, and cuts [9,10]. Numerous pharmaceutical uses were documented for the different extracts of *L. nudicaulis* i.e., antioxidant [10], insecticidal, cytotoxic, antifungal [11] and antimicrobial activity [9]. Chemical classes reported in *L. nudicaulis* include essential oils [9,10], flavonoids, phenolics, alkaloids [12], sesqui-, di-, and triterpenoid/steroids in addition to sphingolipids [13]. We have previously reported the chemical characterization, antioxidant and phytotoxic activities of essential oils of three *Launaea* plants including *L. nudicaulis* [10]. Due to the history of the genus in addition to the potent biological activities of *L. nudicaulis* especially, antioxidant potentiality, existing evidence suggested that it has potential antidiabetic activity [14,15,16,17].

Continuing our goal in exploring the biological potential of Egyptian plants in relation to their chemical profiles [18,19], the present work aimed to (i) determine the antidiabetic effect of *L. nudicaulis* ethanolic extract in streptozotocin (STZ)-induced diabetic (type-1) rats, (ii) study of the potentiality of the extract to act against complications of DM and iii) construct the chemical profile of the extract using UPLC-ESI–qTOF-MS.

## 2. Results and Discussion

### 2.1. Acute Toxicity

Oral administration of a single dose of different concentrations of *L. nudicaulis* EtOH extract (1–3 g/kg body weight) to albino mice groups did not induce any mortality during the first 48 h. After the first 48 h, no mortality was observed during the followed 14 days with these concentrations, compared to negative control. The mortality started with concentration at 4 g/kg/day till 6 g/kg/day. The concentration that killed 50% of animals during the first 48 h was estimated at 5 g/kg/day (Table S1).

### 2.2. Effect on Blood Glucose Levels

Data presented in Table 1 reveals the effect of *L. nudicaulis* ethanol extract on blood glucose levels of STZ- induced diabetic rats and post five weeks, compared to the diabetic control. The extract showed a significant effect on blood glucose level and significantly minimized it during the experimental period, compared to the diabetic control. An increase of the extract doses from 250 mg/kg to 500 mg/kg significantly increased its hypoglycemic effect from 3rd week until the end of the experiment.

Additionally, the time recorded a significant effect on blood glucose levels, compared to the level at the start-time, except the negative control, where time had no significant effect. Blood glucose of diabetic control showed significant elevation compared to the value at the start time. Blood glucose of *L. nudicaulis* extract-treated and standard control groups significantly were decreased along the experimental period to reach the lowest level at the 5th week. Administration of *L. nudicaulis* EtOH extract at two doses for one week significantly decreased blood glucose levels. Continuing the extract administration for another four weeks showed a continuous reduction in blood glucose levels to reach the highest effect at the 5th week, compared to diabetic control. *L. nudicaulis* extract at 500 mg/kg recorded the minimum blood glucose level at 5th week (130.86 ± 5.61 mg/dL), compared to diabetic control (486.00 ± 25.22 mg/kg) at the same time. Blood glucose level of 500 mg/kg treated group at the 5th week (130.86 ± 5.61 mg/dL) was close to that of the negative control (126.20 ± 5.63 mg/dL) at the same time.

### 2.3. Effect on Serum Insulin Levels

**Figure 1:**
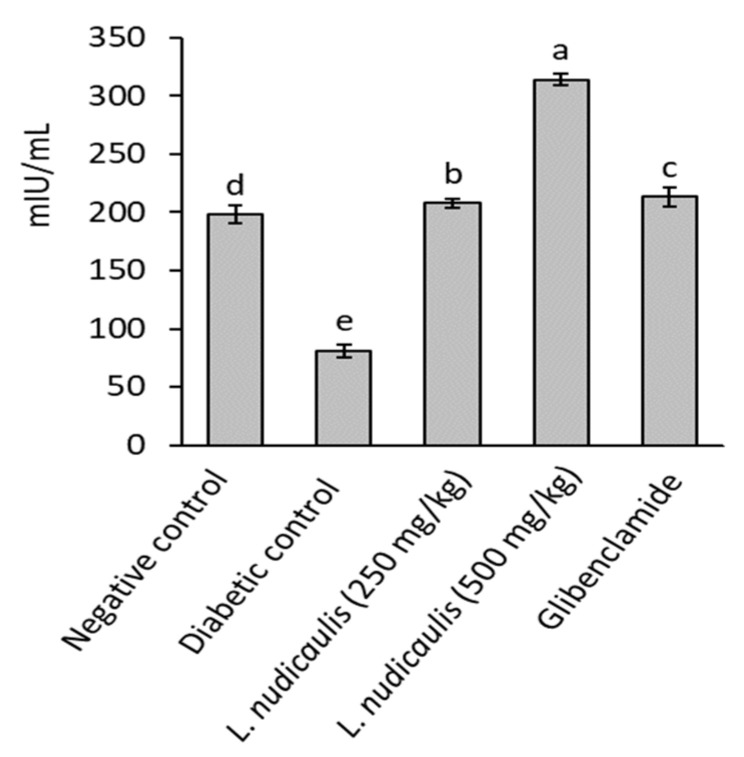
Insulin concentration of STZ- induced diabetic rats treated with *L. nudicaulis* ethanolic extract during 5-weeks. Data are presented as the means ± SE (*n* = 10). Data analyzed by ANOVA one-way. Value with the different superscript letters means significance at a probability level of 0.05.

Diabetic control recorded a significant reduction in serum insulin levels of rats (81.04 ± 5.20 mIU/mL) after 5-weeks, compared to 197.90 ± 7.61 mIU/mL in the negative control (*p* ≤ 0.05). EtOH extract of *L. nudicaulis* at the two doses (250 and 500 mg/kg) significantly maximized serum insulin level of treated groups to 207.78 ± 4.05 and 314.09 ± 5.30 mIU/mL, respectively, with 156.39 and 287.57% increase than that of diabetic control; 81.04 ± 5.20 mIU/Ml (*p* ≤ 0.05) (Figure 1). The extract attained serum insulin close to that recorded in the negative control or slightly higher.

### 2.4. Effect on Rats Weekly Body Weight

Administration of STZ in rats led to a significant decrease in body weight over the time of the experiment (*p* ≤ 0.05) till the end, compared to the rats in the negative control group (Table 2). Plant extract-treated groups and glibenclamide drug showed, in contrast, a restoration in rats’ weight till the end of the experiment. Weekly body weight of the treated groups normally increased from 3-week and continued in significant increase till the 5-week, in comparison with the diabetic control. The effect of *L. nudicaulis* on body weight was similar to the effect of a standard drug.

### 2.5. Effect on Lipid Profile

The diabetic control rats were characterized by disrupted lipid profiles manifested by a significant elevation in TC, TG, and VLDL-C, and LDL-C levels (Table 3), concurrently with a significant reduction in HDL-C level, in comparison to the same parameters in the negative control (*p* ≤ 0.05). Lipid profile biomarkers of diabetic rats were enhanced when rats administrated the extract either at a low or high dose level, compared to the diabetic control. TC, TG, VLDL-C, and LDL-C levels showed significant reductions nearly to the normal levels than that recorded in the negative control.

Also, the HDL-C level was significantly raised and back to its normal level. The risk ratio was also improved as a response to the improvement of lipid profile in the *L. nudicaulis* EtOH extract-treated groups. The effect of the extract on the lipid profile was similar to that of glibenclamide drug in TC, TG and VLDL-C, and LDL-C levels.

### 2.6. Effect on Liver and Kidney Functions

Liver performance of the diabetic control rats was disrupted as evidenced by a significant reduction in total protein production and its fractions; albumin and globulin, concurrent with a significant elevation on AST, ALT, and ALP activities, compared to the corresponding values in the negative control (*p* ≤ 0.05). *L. nudicaulis* EtOH extract returned liver performance of treated-rats towards normalization, where total protein, albumin, and globulin production were significantly raised, concurrently with a significant reduction on AST, ALT, and ALP activities in comparison with the corresponding values on the diabetic control (Table 4). No significant difference was recorded between the effect of a low and high dose of the extract on the liver functions of treated rats, except in albumin and globulin. Compared to the drug glibenclamide, the extract recorded the highest ameliorative effect on liver function

Diabetes induction disrupted renal performance, causing a significant (*p* ≤ 0.05) elevation in creatinine, uric acid, and urea concentrations of the diabetic control rats in comparison with the corresponding values in the negative control (Table 5). *L. nudicaulis* EtOH extract restored renal functions of diabetic rats towards normalization by force-feeding low or high dose, compared to that of the diabetic control. The extract of *L. nudicaulis* caused amelioration on uric acid and urea equal or higher than that caused by glibenclamide.

### 2.7. Effect On Relative Weight of Vital Organs

Data in Table 6 shows the effect of *L. nudicaulis* EtOH extract on the relative weight of vital organs of STZ-induced diabetic rats, in comparison to diabetic control. All vital organs were enlarged as a response to diabetes induction, compared to the negative control, except the pancreas, where it showed atrophy and decreased by 29.73% than that of the negative control. On the other hand, *L. nudicaulis* extract administration for 5-weeks returned all vital organs toward normalization. The relative weight of the pancreas of *L. nudicaulis-* groups was significantly elevated to be within the normal range, compared to the diabetic control. Compared to glibenclamide drug, relative weight of vital organs of *L. nudicaulis-* groups were nearer to that of the negative control.

### 2.8. Effect on Serum Antioxidant and Oxidative Stress Biomarkers

Diabetes induction is associated with remarkable oxidative stress conditions that result in a significant decrease in antioxidant enzyme activities and glutathione concentration, concurrent with a significant elevation in malondialdehyde (MDA, a lipid peroxidation biomarker) levels. Data shown in Table 7 pointed to diabetic control rats were characterized by significant depletion in GSH concentration accompanied with significant decrease in activities of antioxidant enzymes; CAT, GR, GST and GPx, as well as significant increase in MDA content with respect to the negative control values (*p* < 0.05). In comparison with diabetic control results, extract of *L. nudicaulis* showed remarkable antioxidant activity that represented as a significant elevation on GSH level and significant activation on antioxidant enzymes; CAT, GR, GST, and GPx of diabetic rat’s as well as significant depletion on MDA content (*p* < 0.05). The therapeutic effect of *L. nudicaulis* extract on antioxidant enzymes and GSH concentration and MDA content was close to that of the drug glibenclamide.

### 2.9. Histological Examination of Pancreas, Liver, Kidney, and Testis

#### 2.9.1. Pancreas

**Figure 2:**
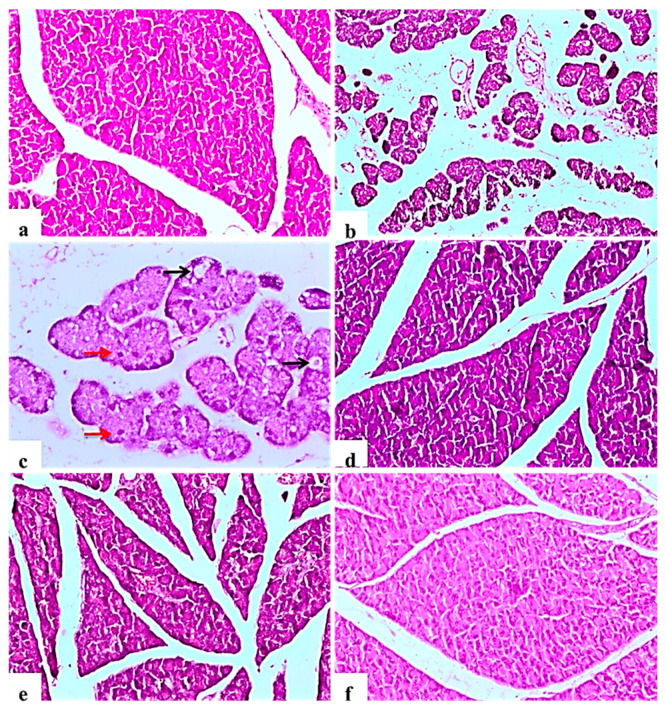
Light photomicrographs of pancreatic tissue (H&E ×100). (**a**): control rat showing normal architecture of the pancreas, acinar cells and arranged into small lobules, pancreatic lobules separated by intact intralobular and interlobular connective tissue septa. (**b**): Subgroup I (diabetic control) showing disorganization of the structure of the endocrine and exocrine cells, shrunken islets with a drastic decrease in the number, necrosis in Langerhans islets, acinar cells were swollen and vacuolated. (**c**): Some islet cells have degenerated and vacuolated (Black arrows), cellular apoptosis (Red arrows), in the form of small pyknotic nuclei and deeply acidophilic cytoplasm (×200). (**d**): Subgroup IV (STZ treated diabetic-rat + glibenclamide) showing normal pancreatic structures and exocrine acini surrounding the islets of Langerhans. (**e**): Subgroup II (STZ treated diabetic-rat + 250 mg ext./kg/day) showing moderate necrotic changes of the pancreas associated with mild reduction in the size and number of the islets of Langerhans. (**f**): Subgroup III (STZ treated diabetic-rat + 500 mg ext./kg/day) showing a remarkable recovery as compared to the STZ treated rats, with mild degree of necrosis of islets of Langerhans.

The negative control rats showed the normal histology of the pancreas without any architectural changes (Figure 2a). In contrast, diabetic control rats showed severe disorganization of the structure of the endocrine and exocrine cells, shrunken islets with a drastic reduction in the number, necrosis in Langerhans islets; acinar cells were swollen and showed small vacuoles (Figure 2b). Some pancreatic islet cells were degenerated and vacuolated, other cells showed cellular apoptosis (Figure 2c). The glibenclamide group showed normal pancreatic structures with exocrine acini surrounding the islets of Langerhans (Figure 2d). *L. nudicaulis* 250 mg group showed moderate necrotic changes of the pancreas associated with a mild reduction in the size and number of the islets of Langerhans (Figure 2e). The effect of *L. nudicaulis* (500 mg/kg) on STZ treated rats group showed a remarkable recovery as compared to the STZ treated rats, with a mild degree of necrosis in islets of Langerhans (Figure 2f).

#### 2.9.2. Kidney

**Figure 3:**
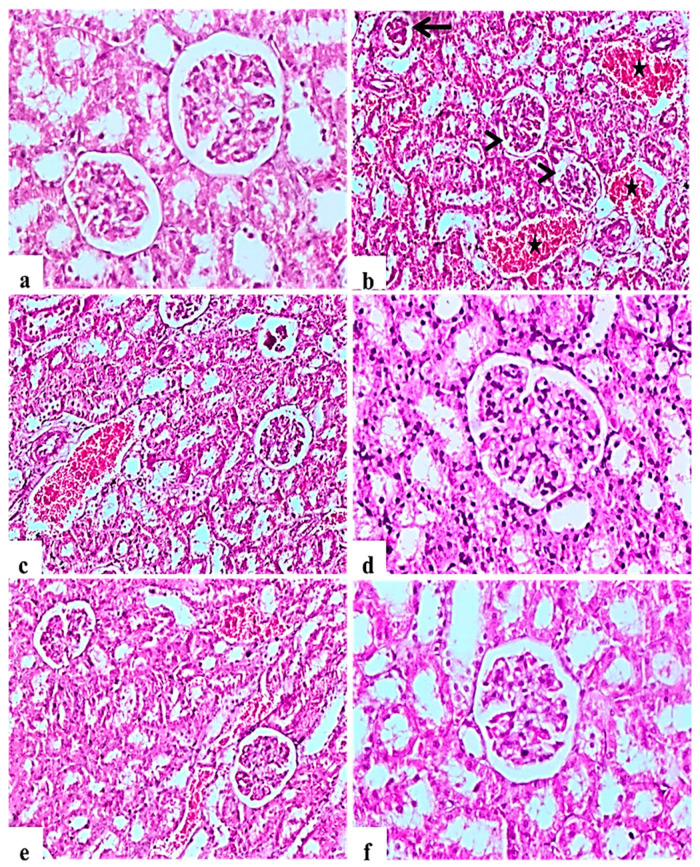
Histological H&E stained sections of Kidney (**a**–**e**) (×200) (**b**,**d**) (×100). a: control untreated rat showing normal glomerulus and renal tubules. b and c: Subgroup I (STZ treated diabetic-rat) rats (STZ treated diabetic-rat) showing atrophy of glomerular tuft (Black arrow), degeneration of renal tubules and increase in the Bowman’ space area (Arrow heads) associated with severe hemorrhage and congestion of renal blood vessels (stars). (**d**): Subgroup IV (STZ treated diabetic-rat + glibenclamide) rats marked amelioration of the glomerular and tubular damage. (**e**): Subgroup II (STZ treated diabetic-rat + 250 mg ext./kg/day) rats showing mild recovery of the pathological picture, still with mild congestion of renal blood vessels. (**f**): Subgroup III (STZ treated diabetic-rat + 500 mg ext./kg/day) rats showing marked recovery and amelioration of the pathological picture associated with the restoration of normal renal architecture.

H&E staining of kidney indicated that the glomerular structure was normal in all negative control rats (Figure 3a). Diabetic control rats showed atrophy of glomerular tuft, degeneration of renal tubules, and increase in the Bowman’ space area associated with severe hemorrhage and congestion of renal blood vessels (Figure 3b,c). *L. nudicaulis* 250 mg group showed moderate necrotic changes with mild congestion of renal blood vessels (Figure 3e). Improvement in the histopathological picture was most noticed in the *L. nudicaulis* 500 mg group, with marked recovery and amelioration of the pathological picture associated with the restoration of normal renal architecture (Figure 3f) close to that of glibenclamide group (Figure 3d).

#### 2.9.3. Liver

**Figure 4:**
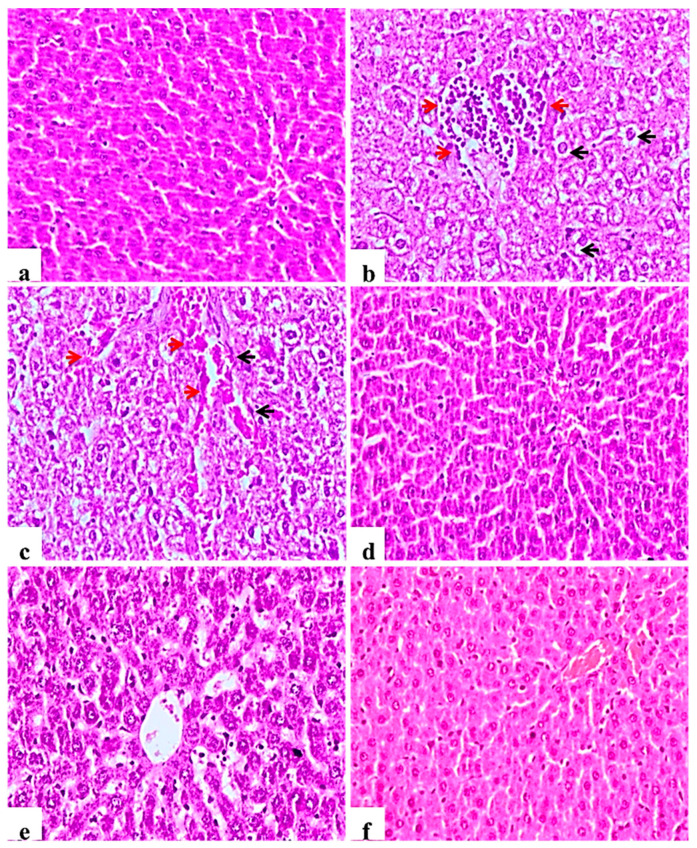
Histological H&E stained sections of liver (×200). (**a**): normal central veins; hepatocytes with the blood sinusoids are present between the hepatic cords. (**b**): Subgroup I (STZ treated diabetic-rat) rats showing mononuclear cell infiltration of the hepatic tissue (Red arrow), Kupffer cell activation, severe vacuolar hydropic degeneration of hepatocytes (Black arrow). (**c**): G1 rats showing Kupffer cell activation (Black arrow), severe vacuolar hydropic degeneration of hepatocytes with severe congestion of central veins and blood sinusoids (red arrow). (**d**): Subgroup IV (STZ treated diabetic-rat + glibenclamide) showing almost normal morphology. (**e**): Subgroup II (STZ treated diabetic-rat + 250 mg ext./kg/day) showing less improvement or restoration of hepatocyte degeneration. (**f**): Subgroup III (STZ treated diabetic-rat + 500 mg ext./kg/day) showing normal architecture with mild vascular congestion.

Examined liver sections of the negative control rats showed normal central veins; hepatocytes with the blood sinusoids were present between the hepatic cords (Figure 4a). In contrast, diabetic control rats showed mononuclear cell infiltration of the hepatic tissue, Kupffer cell activation, severe vacuolar hydropic degeneration of hepatocytes and hyperplasia of bile duct with and loss of hepatic architecture (Figure 4b), other examined sections showed severe congestion of central veins and blood sinusoids (Figure 4c). This picture was markedly ameliorated in the glibenclamide group showing almost normal morphology (Figure 4d). *L. nudicaulis* 250 mg group showed less improvement or restoration of hepatocyte degeneration (Figure 4e), whereas *L. nudicaulis* 500 mg group showed marked amelioration of the pathological picture with normal hepatic architecture.

#### 2.9.4. Testis

**Figure 5:**
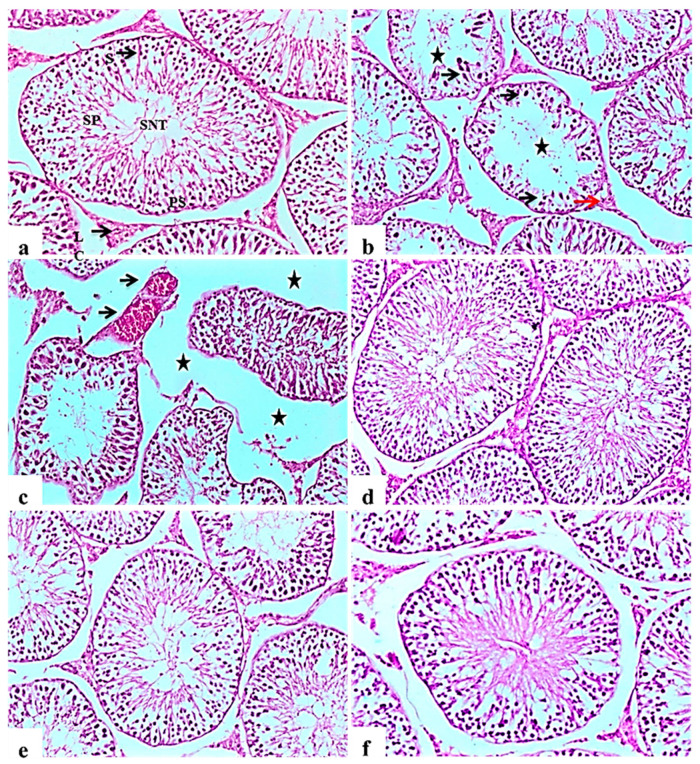
Histological H&E stained sections of the testis (×100). (**a**): control rat showing normal seminiferous tubules architecture (SNT), showing a clear lumen and a normal arrangement of cellular types, lined with series of spermatogenic cells; spermatogonia, primary spermatocytes (PS) and spermatids. Sertoli cells (S) are seen with attached sperms (SP). Interstitial space containing normal Leydig cells (LC). (**b**): Subgroup I (STZ treated diabetic-rat rats showing disorganization and atrophy of seminiferous tubules, absence of spermatogenesis (Star) and severe degenerative change of the lining epithelium Sertoli cells (black arrow). Leydig cells atrophy and vacuolations (red arrow). (**c**): showing disorganization and atrophy of seminiferous tubules, absence of spermatogenesis associated with severe interstitial oedema (Stars) and severe congestion of testicular blood vessels (black arrow). (**d**), (**e**,**f**): Subgroup IV (STZ treated diabetic-rat + glibenclamide, Subgroup II (STZ treated diabetic-rat + 250 mg ext./kg/day) and Subgroup III (STZ treated diabetic-rat + 500 mg ext./kg/day), respectively showing normal structure of seminiferous tubules with mild interstitial oedema, associated with the restoration of spermatogenic cells in most seminiferous tubules.

Histopathological examination of testicular tissue from the negative control rats revealed normal architecture of the seminiferous tubules, showing a clear lumen and a normal arrangement of germinal cells, lined with series of spermatogenic cells; spermatogonia, primary spermatocytes and spermatids. Sertoli cells were observed with attached sperms. Interstitial space containing normal Leydig cells (Figure 5a).

Testes of diabetic control rats showed abnormal pathological alterations, including severe atrophy and disorganization of the seminiferous tubules and severe interstitial oedema, degenerative changes in spermatogenic cells lining the seminiferous tubules, associated with incomplete spermatogenesis. It was observed that the cellular levels of spermatocytes and spermatids showed reduction with the connections between cells intubated. Sertoli cells showed degeneration and necrosis, as well as Leydig cells atrophy and vacuolations (Figure 5b). Some examined sections showed severe disorganization and atrophy of seminiferous tubules accompanied by congestion of testicular blood vessels (Figure 5c). Improvement in the histopathological picture was noticed in all examined sections from diabetic rats in the glibenclamide group revealing apparent normal seminiferous tubules (Figure 5d). *L. nudicaulis* 250 mg group showed a normal structure of seminiferous tubules with moderate interstitial oedema (Figure 5e). Marked improvement in the histopathological picture was noticed in *L. nudicaulis* 500 mg group, where testicular sections showed restoration of spermatogenic cells in most seminiferous tubules with minimal degenerative changes (Figure 5f).

### 2.10. Comprehensive Metabolites Profiling of L. nudicaulis Extract

**Figure 6:**
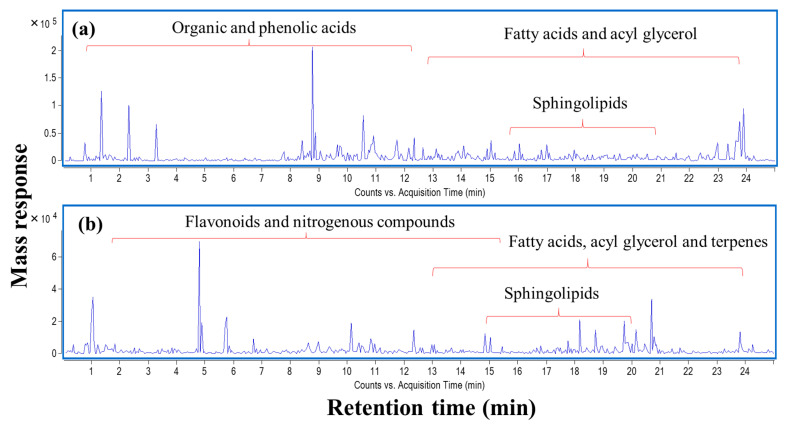
UPLC-qTOF-MS base peak chromatogram of *L. nudicaulis* ethanolic extract detected in (**a**) negative and (**b**) positive ionization modes.

To identify chemical entities within the extract, ultra-high-performance liquid chromatography (UHPLC) coupled to mass spectrometry operated in positive and negative ion modes was carried out providing a comprehensive elution of analytes (Figure 6). To match the solvent polarity of 70% ethanol in water, methanol was chosen as solvent for LCMS analysis. The dielectric constant which reflects solvation power of 100% methanol was reported to be at 33.0 ε, while the respective value of 70% ethanol was 33.5 ε [20]. Further, comparable total phenol contents were obtained from extraction of propolis using methanol and water/ethanol [21]. A total of 113 chromatographic peaks were observed, of which 85 were annotated with fatty acids, acyl glycerol, phenolics, terpenoids, and flavonoids as predominating classes (Table 8).

#### 2.10.1. Flavonoids

Flavonoids are ubiquitous secondary metabolites with known antihyperlipidemic, antitumor, antidiabetic and antioxidant effects [55] Methoxylated flavonoids were characterized by methyl losses (−15 amu) as observed in hydroxymethoxymethylflavan (peak 27; *m*/*z* 271.1311, C_17_H_19_O_3_^+^) yielding fragment ion at *m*/*z* 256. Likewise, a more lypophilic flavone conjugate was found in the peak 75 [M + H]^+^ *m*/*z* 373.1281 with fragment ions at *m*/*z* 358 [M + H − 15]^+^ and 343 [M + H − 30]^+^ annotated as pentamethoxyflavone. Additionally, peak 71 revealed characteristic product ions at *m/z* 212 and 183 ascribed to RDA fission suggesting a dimethoxy B ring and identified as hexamethoxyflavone. Conversely, two free flavonoid aglycones were present in peaks 52 and 56, corresponding to kaempferol (*m/z* 287.0567, C_15_H_11_O_6_^+^) and apigenin (*m*/*z* 271.0627, C_15_H_11_O_5_^+^), respectively.

#### 2.10.2. Simple Phenolics

Simple phenolics were the second dominant class in *L. nudicaulis* after fatty acids/sphingolipids. Phenolic acids *viz*., caffeic acid (peak 44; *m/z* 179.0366) and gallic acid (peak 34; *m/z* 169.0161) displayed a characteristic loss of 44 amu owing to the loss of CO_2_ while caffeic acid esters exhibited a fragment ion at *m/z* 179 in negative ion mode corresponding to the caffeoyl moiety as indicated in caffeoyl tartaric acid (peak 33; *m/z* 311.0438, C_13_H_11_O_9_^−^), *O*-caffeoylquinic acid (peak 32; *m/z* 353.0911, C_16_H_17_O_9_^−^) and caffeic acid ethyl ester (peak 54; *m/z* 207.068, C_11_H_11_O_4_^−^). Another caffeoyl moiety was detected in the peak 23 [M − H]^−^ *m*/*z* 341.0912, annotated as *O*-caffeoylhexose. In the same context, peak 31 demonstrated a molecular ion at *m/z* 309.0283 (C_13_H_9_O_9_^−^) with a caffeoyl moiety and annotated as caffeoyl-*O*-dihydroxy maleic acid. Peak 39 displayed a deprotonated molecular ion at *m/z* 325.0599 with an intense product ion at *m/z* 193 attributed to feruloyl moiety and identified as feruloyl tartaric acid. Furthermore, peak 37 revealed a molecular ion at *m/z* 295.0488 (C_13_H_11_O_8_^−^) with MS^2^ spectrum characterized by a coumaroyl moiety at *m/z* 163 and confirmed its identification as *p*-coumaroyl tartaric acid. Gallic acid and all aforementioned phenolic acid esters are reported for the first time in *L. nudicaulis*.

Four phenolic compounds are also reported here for the first time in *L. nudicaulis*, namely hydroxybutylmethoxyphenol, methoxyphenylacetic acid, methylbatatasin III, and phenethylphenol. Peak 53 [M + H]^+^ *m/z* 197.1188 exhibited product ions at *m/z* 166 and 123 due to the loss of methoxy and hydroxybutyl moieties, respectively annotated as hydroxybutyl-methoxy-phenol. Likewise, methylbatatasin III (peak 20; *m/z* 259.1326, C_16_H_19_O_3_^+^) formed product ions at *m/z* 123 and 108, indicating a methoxy-phenol and methoxy-benzene groups, respectively.

#### 2.10.3. Fatty Acids and Sphingolipids

Nineteen fatty acids encompassing hydroxylated and polyunsaturated forms represent one of the major classes in *L. nudicaulis*. Peak 88 (*m/z* 349.2602, C_18_H_37_O_6_^+^) annotated as tetrahydroxyoctadecanoic acid was the only saturated fatty acid, suggesting a good effect of *L. nudicaulis* extract on human lipid profile. Being precursors of prostaglandins, lipoxin (peak 62; *m/z* 353.2317, C_20_H_33_O_5_^+^), oxolipoxin (peak 66; *m/z* 351.2159, C_20_H_31_O_5_^+^), linolenic acid (peak 89; *m/z* 279.2332, C_18_H_31_O_2_^+^) and its methyl ester (peak 98; *m/z* 293.2487, C_19_H_33_O_2_^+^) exhibited anti-inflammatory effects [56]. Moreover, some hydroxylated fatty acids were present as major peaks and showed additional water molecule loss, aside from their cytotoxic and antimicrobial capacity [57]. For example, hydroxylinolenic acid (293.215, C_18_H_29_O_3_^−^) and hydroxyolinoleic acid (295.2303, C_18_H_31_O_3_^−^) revealed 2 amu mass differences attributed to the extra double bond with fragment ions at *m/z* 275 and 277, respectively due to the subsequent loss of one water molecule. Similarly, compound 63 (309.207, C_18_H_29_O_4_^+^) demonstrated the main MS/MS fragment at *m/z* 273 rationalized by the loss of two water molecules and tentatively characterized as hydroxy-oxo-octadecatrienoic acid. Sphingolipids, detected in peaks 106 (570.5157, C_34_H_68_NO_5_^−^) and 108 (682.6342, C_42_H_84_NO_5_^+^), are involved in several cellular responses viz., autophagy and apoptosis [58]. Peak no. 109 at *m/z* 842.6782 (C_48_H_92_NO_10_^−^) with a characteristic fragment ion at *m/z* 179 indicating a hexose moiety was annotated as iotroridoside. It was previously isolated from a marine sponge *Iotrochota ridley* with potent anti-cancer activity [59]. Another sphingolipid (peak 76; *m/z* 318.2982, C_18_H_40_NO_3_^+^) composed of a long-chain amino alcohol was identified as aminooctadecanetriol.

#### 2.10.4. Amino Acids/Peptides

Being precursors for protein biosynthesis, amino acids and peptides are valuable constituents of food [60]. Acetylated amino acids showed fragment ions attributed to the loss of the acetyl group (−42 amu), as indicated in peak 45 (*m/z* 206.0835, C_11_H_12_NO_3_^−^) with a product ion at *m/z* 164 corresponding to phenylalanine moiety and annotated as *N*-acetyl-L-phenylalanine. Likewise, peak 13 (*m/z* 144.067, C_6_H_10_NO_3_^−^) displayed a loss of acetyl group (*m/z* 102 [M-H-acetyl]^−^) followed by a consequent loss of amino group (*m/z* 87 [M-H-acetyl-NH]^-^) and annotated as *N*-acetylglycine ethyl ester.

#### 2.10.5. Organic Acids

Being eluted earlier due to its high polarity, organic acids of *L. nudicaulis* included quinic (2) and malic (4) acids. Two related organic acids, peaks 24 and 40, with molecular ions [M + H]^+^ *m/z* 229.1443 C_12_H_21_O_4_^+^ and [M − H]^−^ *m/z* 225.1157 C_12_H_17_O_4_^−^ were annotated as hydroxy-dihydrojasmonic and hydroxyjasmonic acids, respectively.

#### 2.10.6. Nitrogenous Compounds

Nitrogenous compounds were detected in positive ion mode due to preferential ionization in that mode, and included aminobenzoic acid (5), dimethylamino-benzaldehyde (12), hydroxybenzylamine (9) and phenylethylamine (22).

In the present study, antidiabetic potentiality of *L. nudicaulis* EtOH extract was evaluated in STZ- induced rats. The hypoglycemic effect of *L. nudicaulis* represented a significant reduction on blood glucose levels concurrent with a significant induction on blood insulin levels as well as a significant amelioration in weekly body gain of rats compared with glibenclamide as a reference drug. The reduction of the blood glucose levels via the effect of the extract induction might be ascribed to its capacity to increase glucose absorption as well as the decreasing of the glucagon releasing (11). All these effects were documented from several medicinal plants including some *Launaea* plants such as *L. taraxacifolia* and *L. cornuta* [61]. Also, the inhibitory action of the extract of α-glucosidase might be one of the possible mechanismatic pathways that increase the polysaccharide conversion to simple sugars [62].

Our findings exhibited that the *L. nudicaulis* extract caused GSH depletion along with potent decreasing of CAT, GR, GST and GPx, as well as significant increasing of MDA content comparing with glibenclamide drug. These results caused decreasing the oxidative stress that manage the diabetic and its associated disorders [3].DM represented dangerous diseases on the liver cells due to the main role of the liver in carbohydrates metabolism. The biochemical analysis exhibited the significant enhancement of all the functions of the liver comparing to the diabetic control group as well as the reference drug, glibenclamide. A significant increasing of total protein, albumin, and globulin as well as decreasing of AST, ALT and ALP were detected by the induction of the two doses of the extract comparing with the negative, glibenclamide and diabetic control groups. Moreover, the *L. nudicaulis* extract was found to improve the renal functions including that deduced via enhancement of creatinine, uric acid, and urea compared with glibenclamide. All these data are in full agreement with Gbadmosi, et al.’s results [61]. All the results revealed to the capability of the extract to manage the serum insulin levels along with the weekly body weight. Nevertheless, observed increase in rats body weight than the negative control group might refer to a side effect that should be more studied in the future.

This activity was also associated with a safety margin that via a significant enhancement of liver, kidney and testis functions along with antioxidant system. Histopathological studies deduced these findings where *L. nudicaulis* extract recovered the alternations in pancreas, liver, kidney, and testis that occurred as a response to diabetes induction. Also, our results revealed that, severe disruption of spermatogenesis in the untreated diabetic rats in comparison with the control, glibenclamide and *L. nudicaulis* treated diabetic rats, as reported in previous studies [63], the cellular levels have been reduced of spermatocytes and spermatids; diabetes has been reported to reduce the number of spermatogenic cells, and the diameter of the seminiferous tubules as a result of oxidative stress and cellular apoptosis [64]. Sertoli cells showed degeneration and necrosis as well as atrophy and vacuolations of Leydig cells because of the alterations in pituitary gonadotropins, depression of synthesis and secretion of testosterone by Leydig cells [65] and the stimulated expression of Caspase12, Grp78, and Chop., inducing cell cycle arrest and apoptosis of Leydig cells [66]. These severe histopathological alterations were, however, ameliorated by extract of *L. taraxacifolia*, this marked improvement is attributed to its flavonoids enriched contents, as previously documented by Adejuwon, et al. [67]. Moreover, Khan [68] suggested that *L. procumbens* extract has a great capability to protect testis against testicular oxidative damages, possibly credited to its antioxidant effects of its bioactive compounds. The improvement in the histopathological picture noticed in all examined sections from *L. nudicaulis* groups which is attributed to lowering of the elevated blood glucose levels [69], the significant hypoglycemic effect that agreed with the present findings that treated diabetic rats with *L. nudicaulis* and reduced histopathological alterations due to the antioxidant nature as well as decreasing of apoptosis β-cells and oxidative stress reduction [70]. The islet cell injury, particularly β-cell damage is attributed to the increased oxidative stress, metabolic stress, increased endoplasmic reticulum stress, activation of inflammatory pathways, and the hyperglycemia; hyperlipidemia causing cellular apoptosis [71,72]. Glibenclamide- and *L. nudicaulis*-treated diabetic rats showed an improvement in the islet cell morphology with still minimal apoptotic changes and reduction in the size and number of the islets of Langerhans. These findings were consistent with the results of Erejuwa, et al. [73] who showed that glibenclamide preserved beta-cell mass and morphology in diabetic rats. Khan, et al. [74] found that *L. procumbens* protected the antioxidant machineries of the pancreas, due to phenolic and polyphenolic compounds as well as its marked protection against DNA damage. All these results revealed a potent hypoglycemic effect with a reasonable margin of safety, qualifying it as a drug for controlling diabetes.

The biological potentialities of the medicinal plants are directly correlated with their secondary metabolites. According to this fact, the significant role of *L. nudicaulis* ethanolic extract on diabetes mellitus and its comorbidities in diabetic control rats was established upon the metabolites of the plant. The chemical profile revealed that is rich with several metabolite classes including flavonoids, phenolics, fatty acids, sphingolipids, amino acids, peptides, organic acids, and nitrogenous compounds. All these classes of metabolites were already described to exert biological activities against hepatic damage and toxicity [19,75], hyperlipidemia [76], diabetes [19], and gastric ulcers [8,77].

The polyphenolics in *L. nudicaulis* including phenolic acids and flavonoids, were reported to have potential role in the inhibition and/or controlling of diabetes via several pathways such as (i) pancreatic islet β-cell protection, (ii) inhibition and/or decrease of β-cell apoptosis, (iii) increased β-cell proliferation, (iv) oxidative stress reduction, (v) insulin secretion activation, (vi) digestive enzymes and glucose absorption inhibition, and other effects [70]. Additionally, the roles of these phenolic compounds in the enhancement of diabetic complications were reported [61] and help account for the protective effects observed in this study. The simple phenolic acids i.e., gallic, caffeic, and *p*-coumaric acids and their derivatives were described as significant hypoglycemic agents [78,79], which act through multiple mechanisms i.e., inhibition of gluconeogenesis, increasing of C-peptide and insulin secretion and glycolysis, enhancing of the functions of liver and renal in addition of others [70].

Likewise, flavonoids have been described as potential hypoglycemic agents that may act via several mechanisms including: (i) enhancement of activated cellular protein expression of the adenosine monophosphate-activated protein (AMP), (ii) reduction of the apoptosis of the cellular via caspase 3 deactivation, (iii) activation of the insulin secretion and production from β-cells, (iv) increasing of cells absorption of the glucose, and (v) activation of the new glucose carriers synthesis [70,80,81].

Nobiletin, a hexamethoxyflavone, demonstrated antidiabetic and anti-inflammatory activities [82]. Moreover, tangeretin, a pentamethoxyflavone, improves secretion of insulin and decreases blood glucose via stimulating glucose uptake [83]. Compared to phenolic acids and flavonoids, relatively polar metabolites, non-polar constituents such as sphingolipids also detected in this study are recorded as potential antidiabetic agents [84]. Sphingolipids acted via numerous pathways comprising the inauguration of apoptosis of β-cell, insulin resistance, and decreasing of the insulin gene expression [84].

The chemical profiling suggests that as typical in most plant extracts, antidiabetic action in *L. nudicalis* is more likely mediated via a synergized action of metabolites for enhancement of antidiabetic activity of *L. nudicalis*.

## 3. Materials and Methods

### 3.1. Plant Materials Collection, Identification, and Extract Preparation

The aerial parts of *L. nudicaulis* were collected from Wadi Hagul, Eastern Desert of Egypt (30°02′34.3″ N 32°05′40.6″ E), during the flowering stage in April 2018. The identification of the plant was performed by Dr. Ahmed M. Abd-ElGawad, Associate professor of plant ecology, Faculty of Science, Mansoura University, Mansoura, Egypt, according to Boulos [85] and Tackholm [86]. A plant voucher specimen (Mans.001121406) was deposited in the herbarium of Botany Department, Faculty of Science, Mansoura University, Mansoura, Egypt.

The collected plant materials were dried in air and shade condition for ten days. After complete dryness, the sample (760 g) was ground into a fine powder, extracted with a mixture of H_2_O-EtOH (3:7, 3 L) at room temperature (25 ± 2 °C), and then filtered. The extraction procedure was performed three times. All the extracts were collected and dried under reduced pressure to afford black gum (28.7 g). The extract was stored at 4 °C in the deep freezer until further chemical and biological analyses.

### 3.2. Chemicals

Streptozotocin were purchased from Sigma-Aldrich Company (St. Louis, MO, USA). the diabetic drug glibenclamide (Daonil^®^) was obtained from Sanofi Aventis (Cairo, Egypt). All kits were obtained from Biodigonestic Diagnostics Egypt (Dokki, Giza, Egypt). All used chemicals were analytical grade. Ethanol (99%) was purchased from El-Naser Company for Intermediate Chemicals (Cairo, Egypt).

### 3.3. Experimental Animals

Healthy adult male albino rats of Wistar strain (160–200 g weight 60–80 day age), were obtained from the central animal house of National Research Centre (Dokki, Giza, Egypt). The central animal house conditions were well ventilated, 20 to 25 °C temperature, 50–55% of relative humidity and 12 h dark/light cycle. Rats were kept in plastic hygienic cages during the experimental period (6 weeks). Pellet diet was obtained from the central animal house of National Research Centre. The diet contains 4.60% fat, 25.00% crude protein, 4.78% crude fiber and 6.71% crude ash (the diet composition was analyzed according to AOAC [87]. Pellets and water were *ad libitum*. The study was conducted based upon the rules of the ethics committee of the National Research Centre and in accordance with the Guide for the Care and Use of Laboratory Animals of the National Institutes of Health in compliance with the guidelines from the Canadian Council on Animal Care (approval no: 19-074).

### 3.4. Acute Toxicity (LD_50_)

Acute toxicity of the EtOH extract of *L. nudicaulis* was performed according to per OECD guideline 4235 [88] (2001). Dosing pattern started from 1 g/kg as a single dose and increased to reach 6 g/kg body weight orally, while the control group received only normal saline. Mortality in all groups (8 mice each) was observed for 48 h for assessing toxicity. Alive animals were observed for a period of two weeks. Using mortality number in each concentration during the first 48 h and BioStat program (BioStat 2009 Build 5.8.4.3 © 2021 analystSoft Inc., Alexandria, VA, USA), the extract dose that killed 50% of the animals (LD_50_) was estimated at 5 g/kg.

### 3.5. Hypoglycemic Experiment

#### 3.5.1. Diabetes Induction

Wistar-albino male rats (160–200 g) were intraperitoneally i.p. injected with one dose of streptozotocin (STZ). Overnight fasted rats were injected with freshly STZ in 0.1 M citrate buffer (pH 4.5) at a dose of 55 mg/kg. STZ- injected rats were administrated glucose (25%) after one hour of STZ injection, and they were kept on glucose (5%) overnight to overcome STZ-induced hyperglycemia [89]. Diabetes was presented by the presence of hyperglycemia, polyuria, and body weight loss. After seven days from STZ-injection, fasting blood glucose levels were measured for all STZ animals. Animals with blood glucose levels above 250 mg/dL, were considered as diabetic and were selected for subsequent experiments [4].

#### 3.5.2. The *L. nudicaulis* Extract and Standard Drug Dosing Protocol

According to Ghosh [90] and Garg, et al. [91], two doses of 500 and 250 mg/kg body weight were selected to assess the anti-hyperglycemic effect of the ethanolic extract of *L. nudicaulis* at 10th and 20th of the LD_50_ value. The anti-hyperglycemic effect was evaluated by comparing it with the effect of the standard drug glibenclamide. The glibenclamide was tested at its recommended dose of 5 mg/kg/day [92]. Animals administered both extract and glibenclamide dissolved in 1.0 mL normal saline (0.9%) orally using a stomach tube.

#### 3.5.3. Experimental Design

Seventy male rats were divided into two main groups. 1st group was non-diabetic rats (ten rats), were injected with 1.00 mL of 0.1 M citrate buffer (pH 4.5) only and served as a negative control for five weeks. The 2nd group was the diabetic group (60 rats), rats were injected with a single dose of STZ at 55 mg/ kg in citrate buffer, and after one week, fasting blood glucose levels were measured for all animals in this group, and animals with the highest blood glucose (above 250 mg/dL) levels were selected for subsequent experiments (44 rats). Then, the 2nd group, Forty- four diabetic male albino rats, was divided into four subgroups: Subgroup I: diabetic rats (14 rats) administrated saline for five weeks, and was kept as diabetic control. Subgroup II: diabetic rats (10 rats), administrated ethanolic extract of *L. nudicaulis* at 250 mg/kg body weight/day for five weeks. Subgroup III: diabetic rats (10 rats), administrated with ethanolic extract of *L. nudicaulis* at 500 mg/kg body weight/day for five weeks. Subgroup IV: diabetic rats (10 rats), administrated with glibenclamide at a recommended dose of 5 mg/kg body weight/day for five weeks.

Using a digital balance, rats were weighed at the start of the experiment, and every week for five weeks. After five weeks (experimental period), rats were anesthetized by injection of 87 mg/kg of body weight and xylazine 13 mg/kg dissolved in normal saline and each rat i.p. simultaneous received 0.2 mL/100 g body weight [93]. Under anesthesia, blood samples were collected from the retro-orbital plexus using capillary tube. Sera were separated by centrifugation (4000× *g* and 10 min using a Sigma Labor Zentrifugen). Organs were collected, washed with ice saline solution, and then weighted freshly. A piece of pancreas, liver, kidney, and testis from each rat was kept immediately in formalin 10% for histopathological examination.

#### 3.5.4. Measurement of Blood Glucose Levels

Every week during the experimental period, rats fasted overnight, and blood samples were collected from the tip of tail veins of each rat. The blood sample were collected in the morning (at 9 o’clock) prior to administering the extract or eating food to determine the fasting blood glucose level. Based on the glucose oxidase method, glucose concentration was estimated in whole blood samples immediately after collection, using Gluco Star Test Strip (Taidoc Technology Corp., New Taipei, Taiwan). The change percentage in each row calculated as:

Change% = [(concentration at start time- concentration at 1st week)/concentration at start time] × 100

#### 3.5.5. Determination of Serum Insulin Level and Serum Lipid Profile

Serum insulin level was measured using rat insulin ELISA kits from BioVision Incorporated (Milpitas, CA, USA). Lipid profile including; total cholesterol (TC), high-density lipoprotein cholesterol (HDL-C), and triglycerides (TG) were estimated according to Allain, et al. [94], Naito [95], and Fossati and Prencipe [96], respectively. Low-density lipoprotein cholesterol (LDL-C), very low-density lipoprotein cholesterol (VLDL-C), and risk ratio were calculated according to Naito [95], Friedewald, et al. [97], and Kikuchi-Hayakawa, et al. [98], respectively.

#### 3.5.6. Determination of Liver and Kidney Functions

Liver function parameters; total protein, albumin and liver enzymes activities; alkaline phosphatase (ALP), aspartate aminotransferase (AST) and alanine aminotransferase (ALT), were measured spectrophotometrically according to Henry [99], Doumas, et al. [100], Belfield and Goldberg [101], and Rettman and Frankel [102], respectively. Globulin was calculated by the difference between total protein and albumin [103]. Kidney function parameters; urea, uric acid, and creatinine, were estimated spectrophotometrically as methods described by Tabacco, et al. [104], Gochman and Schmitz [105], and Faulkner and King [106], respectively.

#### 3.5.7. Determination of Antioxidant and Oxidative Stress Biomarkers of Serum

The malondialdehyde (MDA) was usually used as oxidative stress parameters. The MDA was determined spectrophotometrically following the method of Ohkawa, et al. [107]. Non enzymatic antioxidant, reduced glutathione concentration (GSH) was determined in serum according to the method of Griffith [108]. Enzymatic antioxidants parameters including glutathione reductase (GR), Glutathione-S-transferase (GST), Glutathione peroxidase (GPx), and catalase (CAT) activities were determined spectrophotometrically in serum according to Goldberg and Spooner [109], Paglia and Valentine [110], Habig, et al. [111], and Beers and Sizer [112], respectively.

### 3.6. Histopathological Examination

The testis, pancreas, kidney and liver specimens were instantly dissected out, excised, and fixed in 10% neutral buffered formalin fixative solution, dehydrated and embedded in paraffin; solid sections of 4–5 μm. The sections were stained with hematoxylin–eosin (H & E) and followed by investigation using a light microscope (CX 41, Olympus, Tokyo, Japan) [113].

### 3.7. High-Resolution Ultra-Performance Liquid Chromatography-Mass Spectrometry Analysis (UPLC-ESI–qTOF-MS)

UPLC-MS analysis was performed following exact conditions described in Farrag, et al. [8]. Briefly, dried finely pulverized *L. nudicaulis* specimen (10 mg) was extracted by adding 2 mL 100% MeOH, containing 10 μg/mL^−1^ umbelliferone as an internal standard sonicated for 20 min with frequent shaking, then centrifuged at 12,000× *g* for 10 min to remove debris. The filtered extract through a 22-μm filter (about 500 μL) was subjected to solid-phase extraction using a C_18_ cartridge as previously described [114]. *L. nudicaulis* extract (2 μL) was loaded on HSS T3 column (100 × 1.0 mm, particle size 1.8 μm; Waters) installed on an ACQUITY UPLC system (Waters, Milford, MA, USA) equipped with a 6540 Ultra-High-Definition (UHD) Accurate-Mass Q-TOFLC/MS (Agilent, Palo Alto, CA, USA) coupled to an ESI interface, operated in positive or negative ion mode following conditions described in 113. Characterization of compounds was performed by the generation of the candidate formula with a mass accuracy limit of 10 ppm, and also considering RT, tandem MS2 data, and searching reference literature and the Phytochemical Dictionary of Natural Products Database. Peaks were detected in both negative and positive (deviating values in brackets) ion modes.

### 3.8. Statistical Analysis

Data were expressed as mean ± SE (standard error) for 10 rats. Variables of blood glucose concentration and body weight were statistically analyzed by two-way ANOVA followed by a Duncan’s hoc test, was used to compare multiple groups, and all comparisons were significant when *p* ≤ 0.05 using software COSTAT (version 6.400, Cohort Software, Birmingham, UK). Other results were statistically analyzed by one-way ANOVA followed by a by a Duncan’s hoc test, was used to compare multiple groups, and all comparisons were significant when *p* ≤ 0.05 using software COSTAT.

## 4. Conclusions

Our study provides the first insights into the antidiabetic potential of *L. nudicaulis* alcoholic extract in STZ-induced diabetic rats as revealed via biochemical and histopathological examinations. The extract reduced blood glucose levels and increased the serum insulin levels of diabetic control rats. Additionally, the extract prevented diabetic complications, including loss of weight, liver and kidney disruption and oxidative stress. Whether the anti-diabetic effect of *L. nudicaulis* ehtanolic extract is attributable to the action of a single chemical or the synergy of multi-components as typical in phytomedicines has yet to be confirmed using isolated compound bioassays. The myriad of chemical classes identified in extract including flavonoids, phenolics, acyl glycerols and nitrogenous compounds, several of which are reported antidiabetic agents suggests more for a synergized action and should be the next logical step. Fractionation and or isolation of the crude extract should help confirm such hypothesis.

## Figures and Tables

**Table 1:** Glucose level (mg/dL) of STZ- induced diabetic rats treated with *L. nudicaulis* ethanolic extract during 5-weeks.

Treatments	Blood Glucose Level (mg/dL)					
	Start Time	1st Week	2nd Week	3rd Week	4th Week	5th Week
Negative control	124.0 ± 6.12 ^o^	126.6 ± 7.77 ^o^	127.6 ± 8.32 ^o^	123.8 ± 7.92 ^o^	125.8 ± 7.46 ^o^	126.2 ± 5.63 ^o^
		0.2% *	2.9%	−0.2%	1.5%	1.8%
Diabetic control	411.9 ± 17.53 ^d^	434.4 ± 15.19 ^c^	467.2 ± 15.56 ^b^	485.9 ± 17.17 ^a^	486.3 ± 20.07 ^a^	486.00 ± 25.22 ^a^
		5.5%	13.42%	18.0%	18.0%	18.0%
*L. nudicaulis* (250 mg/kg/day)	408.5 ± 8.86 ^d^	302.5 ± 17.23h ^h^	254.1 ± 11.48 ^k^	225.9 ± 11.47 ^l^	217.8 ± 10.28 ^l^	188.4 ± 7.71 ^m^
		−26.0%	−37.8%	−44.7%	−46.70%	−53.9%
*L. nudicaulis* (500 mg/kg/day)	410.0 ± 9.13 ^d^	364.4 ± 11.97 ^f^	263.6 ± 14.67 ^j^	194.6 ± 9.41 ^m^	161.1 ± 4.85 ^n^	130.9 ± 5.61 ^o^
		−11.1%	−35.7%	−52.5%	−60.7%	−68.1%
Glibenclamide (5 mg/kg/day)	408.7 ± 5.71 ^d^	380.7 ± 17.38 ^e^	354.7 ± 10.72 ^g^	289.9 ± 14.16 ^i^	244.7 ± 9.76 ^k^	198.4 ± 7.87 ^m^
		−6.9%	−13.2%	−29.1%	−40.1%	−51.5%

Data presented as mean ± SE (*n* = 10). Data were analyzed using ANOVA two-way followed with post hoc for multiple comparisons. The means followed by the same letter are not significantly different from each other at 5% probability level (Duncan s multiple range test). * means the change (%) = ((glucose level at the start time—glucose level of the 1st week)/glucose level at the start time) ×100.

**Table 2:** Body weight (g) of STZ- induced diabetic rats treated with *L. nudicaulis* ethanolic extract for 5-weeks.

Time	Start Time	1-Week	2-Weeek	3-Week	4-Week	5-Week
Negative control	167.0 ± 4.97 ^g,h^	180.4 ± 7.12 ^f^	190.0 ± 5.30 ^d^	197.8 ± 3.71 ^c^	212.4 ± 5.15 ^b^	227.0 ± 3.92 ^a^
		+13.4 *	+9.6	+7.8	+14.6	+14.6
Diabetic Control	166.1 ± 4.36 ^g,h^	153.8 ± 3.69 ^j,k^	138.8 ± 5.80 ^m,n^	133.4 ± 3.61 ^n^	123.4 ± 1.80 ^o^	107.5 ± 1.67 ^p^
		−12.3	−15.0	−5.4	−10.0	−15.5
*L. nudicaulis* 250 mg/kg	150.0 ± 13.30 ^k,l^	134.1 ± 1.51 ^n^	147.0 ± 4.97 ^l^	161.2 ± 9.66 ^h,i^	167.0 ± 7.42 ^g^	196.2 ± 11.22 ^c^
		−15.9	+12.9	+14.2	+5.8	+29.2
*L. nudicaulis* 500 mg/kg	161.0 ± 11.81 ^g,h,i^	150.1 ± 8.28 ^k,l^	156.9 ± 6.20 ^i,j^	165.7 ± 6.37 ^g,h^	183.9 ± 7.45 ^e,f^	210.2 ± 9.12 ^b^
		−10.9	+6.8	+8.9	+18.2	+26.4
gilbenclamide 5 mg/kg/day	162.0 ± 7.47 ^g,h,i^	140.0 ± 6.73 ^m^	153.0 ± 10.21 ^j,k^	167.0 ± 3.08 ^g,h^	181.3 ± 6.39 ^f^	189.8 ± 3.40 ^d,e^
		−22.0	+13.0	+14.0	+14.3	+8.5

Data are presented as the means ± SE (*n* = 10). Data of wight analyzed by ANOVA two-way. Value with different letters has significant variation at *p* < 0.05. * indicates weekly body weight gain = body weight of week—body weight of the previous week.

**Table 3:** Lipid profile of STZ- induced diabetic rats treated with *L. nudicaulis* ethanolic extract for 5-weeks.

Groups	TC (mg/dL)	HDL-C (mg/dL)	TG (mg/dL)	VLDL-C (mg/dL)	LDL-C (mg/dL)	Risk Ratio %
Negative control	82.30 ± 7.33 ^c^	49.10 ± 2.91 ^a^	142.87 ± 2.85 ^c^	28.57 ± 0.57 ^c^	5.09 ± 0.94 ^d^	0.11 ± 0.01 ^d^
Diabetic control	194.86 ± 10.65 ^a^	34.45 ± 1.40 ^c^	232.90 ± 8.43 ^a^	46.58 ± 1.69 ^a^	113.83 ± 6.75 ^a^	3.33 ± 0.25 ^a^
*L. nudicaulis* 250 mg/kg/day	88.29 ± 6.88 ^b^	44.96 ± 1.61 ^b^	148.26 ± 7.72 ^b^	29.65 ± 1.54 ^b^	13.68 ± 0.94 ^c^	0.31 ± 0.03 ^c^
*L. nudicaulis* 500 mg/kg/day	92.28 ± 5.25 ^b^	44.20 ± 2.46 ^b^	136.08 ± 7.35 ^d^	27.22 ± 1.47 ^d^	20.88 ± 1.43 ^b^	0.47 ± 0.04 ^b^
Glibenclamide 5 mg/kg/day	94.54 ± 5.00 ^b^	46.05 ± 3.12 ^b^	150.18 ± 4.81 ^b^	30.05 ± 0.96 ^b^	18.60 ± 1.90 ^b^	0.41 ± 0.03 ^b^

Values represent means ± SE (*n* = 10). Means with different letters within the same column are significantly different *p* ≤ 0.05.

**Table 4:** Liver functions of STZ-induced diabetic rats treated with *L. nudicaulis* ethanolic extract during 5-weeks.

Groups	Total Protein g/dL	Albumin g/dL	Globulin g/dL	AST U/L	ALT U/L	ALP U/L
Negative control	7.39 ± 0.44 ^a^	4.08 ± 0.34 ^c^	3.31 ± 0.64 ^a^	57.35 ± 4.47 ^c^	31.08 ± 3.77 ^c^	64.84 ± 36.80 ^c^
Diabetic control	4.81 ± 0.26 ^b^	2.91 ± 0.13 ^d^	1.89 ± 0.32 ^c^	119.56 ± 8.87 ^a^	84.41 ± 6.16 ^a^	95.84 ± 3.40 ^a^
*L. nudicaulis* 250 mg/kg/day	7.15 ± 0.65 ^a^	5.21 ± 0.23 ^a^	1.94 ± 0.66 ^c^	86.23 ± 3.89 ^b^	42.60 ± 3.40 ^b^	75.20 ± 0.79 ^b^
*L. nudicaulis* 500 mg/kg/day	7.22 ± 0.54 ^a^	4.66 ± 0.40 ^b^	2.57 ± 0.83 ^b^	82.19 ± 6.36 ^b^	36.21 ± 3.11 ^b^	71.49 ± 0.44 ^b^
Glibenclamide 5 mg/kg/day	7.19 ± 0.55 ^a^	4.81 ± 0.28 ^b^	2.39 ± 0.34 ^b,c^	91.99 ± 5.20 ^b^	44.48 ± 2.45 ^b^	71.35 ± 0.76 ^b^

AST: aspartate aminotransferase; ALT: alanine aminotransferase; ALP: Alkaline phosphatase; Values represent means ± SE (*n* = 10). Means with different letters within the same column are significantly different *p* ≤ 0.05.

**Table 5:** Renal functions of STZ- induced hyperglycemic rats treated with *L. nudicaulis* ethanolic extract.

Groups	Creatinine mg/dL	Uric Acid mg/dL	Urea mg/dL
Negative control	1.21 ± 0.02 ^d^	4.11 ± 0.08 ^d^	4.32 ± 0.05 ^e^
Diabetic control	2.42 ± 0.08 ^a^	11.91 ± 0.30 ^a^	8.29 ± 0.09 ^a^
*L. nudicaulis* 250 mg/kg/day	1.71 ± 0.08 ^b^	7.10 ± 0.20 ^b^	5.42 ± 0.12 ^c^
*L. nudicaulis* 500 mg/kg/day	1.64 ± 0.05 ^b^	5.10 ± 0.14 ^c^	4.91 ± 0.10 ^d^
Glibenclamide 5 mg/kg/day	1.41 ± 0.04 ^c^	6.89 ± 0.14 ^b^	6.55 ± 0.17 ^b^

Values represent means ± SE (*n* = 10). Means with different letters within the same column are significantly different *p* ≤ 0.05.

**Table 6:** Relative weight of organs of STZ- induced diabetic rats treated with *L. nudicaulis* ethanolic extract.

Groups	Liver	Kidney	Spleen	Brain	Pancreas	Heart	Lung	Testes
Negative control	4.14 ± 0.26 ^c,d^	0.84 ± 0.05 ^c^	0.67 ± 0.07 ^b^	0.80 ± 0.10 ^b,c^	0.37 ± 0.07 ^b^	0.37 ± 0.04 ^b^	0.74 ± 0.10 ^b,c^	1.31 ± 0.15 ^c^
Diabetic control	6.23 ± 0.16 ^a^	1.13 ± 0.04 ^a^	0.75 ± 0.05 ^a^	1.09 ± 0.08 ^a^	0.26 ± 0.08 ^c^	0.45 ± 0.09 ^a^	1.00 ± 0.22 ^a^	1.95 ± 0.28 ^a^
*L. nudicaulis* 250 mg/kg/day	4.84 ± 0.14 ^b^	0.97 ± 0.19 ^b^	0.57 ± 0.13 ^c^	0.8 ± 0.29 ^b,c^	0.47 ± 0.04 ^a^	0.38 ± 0.06 ^b^	0.85 ± 0.23 ^b^	1.60 ± 0.04 ^b^
*L. nudicaulis* 500 mg/kg/day	4.29 ± 0.37 ^c^	0.79 ± 0.05 ^c^	0.52 ± 0.11 ^c^	0.76 ± 0.02 ^c^	0.36 ± 0.06 ^b^	0.36 ± 0.04 ^b^	0.70 ± 0.03 ^c^	1.29 ± 0.03 ^c^
Glibenclamide 5 mg/kg/day	4.06 ± 0.26 ^d^	1.01 ± 0.10 ^b^	0.57 ± 0.09 ^c^	0.93 ± 0.13 ^b^	0.37 ± 0.04 ^b^	0.45 ± 0.08 ^a^	0.78 ± 0.04 ^b,c^	1.47 ± 0.24 ^b^

Values represent means ± SE (*n* = 10). Means with different letters within the same column are significantly different *p* ≤ 0.05.

**Table 7:** Serum malondialdehyde and antioxidant biomarkers of STZ- induced hyperglycemic rats treated with *L. nudicaulis* ethanolic extract.

Groups	MDA nmol/mL	CAT (U/L)	GSH (mg/dL)	GR (µmol/mg protein/min)	GST (µmol/mg protein/min)	GPx (µmol/mg protein/min)
Negative control	3.54 ± 0.31 ^e^	118.82 ± 7.77 ^a^	4.70 ± 0.24 ^c^	5.80 ± 0.29 ^c^	3.91 ± 0.20 ^d^	2.40 ± 0.12 ^b^
Diabetic control	10.414 ± 0.23 ^a^	86.96 ± 9.26 ^d^	1.76 ± 0.20 ^d^	1.86 ± 0.21 ^d^	1.32 ± 0.15 ^e^	0.80 ± 0.09 ^c^
*L. nudicaulis* 250 mg/kg/day	6.43 ± 0.33 ^b^	108.89 ± 4.49 ^b,c^	4.682 ± 0.09 ^c^	5.92 ± 0.12 ^c^	4.11 ± 0.08 ^c^	2.26 ± 0.06 ^b^
*L. nudicaulis* 500 mg/kg/day	4.46 ± 0.31 ^d^	113.46 ± 5.47 ^a,b^	5.22 ± 0.14 ^a^	6.89 ± 0.18 ^a^	4.34 ± 0.20 ^b^	2.78 ± 0.07 ^a^
Glibenclamide 5 mg/kg/day	5.02 ± 0.36 ^c^	103.20 ± 6.04 ^c^	4.94 ± 0.29 ^b^	6.37 ± 0.37 ^b^	5.40 ± 0.31 ^a^	2.67 ± 0.14 ^a^

Values represent means ± SE (*n* = 10). Means with different letters within the same column are significantly different *p* ≤ 0.05. MDA; malondialdehyde; GSH; glutathione-l-reduced, GR; glutathione reductase, GST; glutathione-S-transferase, GPx; glutathione peroxidase, and CAT; catalase.

**Table 8:** Identified metabolites in *L. nudicaulis* alcoholic extract *via* UPLC-qTOF-MS.

No.	RT	Formula	Name	Class	Precursor	Fragmentation	Error	Ref.
1	1.088	C_5_H_9_O_5_^−^	Pentose	Carbohydrate	149.0453	101, 89, 75, 73	1.85	
2	1.179	C_7_H_11_O_6_^−^	Quinic acid	Organic acid	191.0574	127, 105, 85, 75	−7.12	[22]
3	1.225	C_7_H_14_NO_2_^+^	Unknwon	Nitrogenous compound	144.1025	98, 84, 70	2.47	
4	1.271	C_7_H_8_NO_2_^+^	Aminobenzoic acid	Nitrogenous compound	138.0546	92, 78, 67	2.39	[23]
5	1.271	C_4_H_5_O_5_^−^	Malic acid	Organic acid	133.0156	115, 75, 73, 71	−9.73	[24]
6	1.476	C_10_H7O_4_^−^	Scopoletin	Coumarin	191.0368	-	9.51	[25]
7	1.636	C_11_H_16_NO_8_^−^	Deoxy-dehydro-*N*-acetylneuraminic acid	Organic acid	290.0898	200, 128	−5.61	[26]
8	1.865	C_12_H_19_N_2_O_7_^−^	Unknown	Pyrazine	303.1216	213, 123, 87	−7.28	[27]
9	2.642	C_7_H_10_NO^+^	Hydroxybenzylamine	Nitrogenous compound	124.0753	108, 94	5.8	[28]
10	2.733	C_6_H_14_NO_2_^+^	Leucine	Amino acid	132.1029	86, 69	−6.85	[29]
11	2.779	C_17_H_19_O_4_^+^	Unknown	Phenolic acid	287.127	229, 193, 175	−2.73	
12	3.098	C_9_H_12_NO^+^	Dimethylamino-benzaldehyde	Nitrogenous compound	150.0904	135, 108	7.83	
13	3.19	C_6_H_10_NO_3_^−^	*N*-Acetylglycine ethyl ester	Amino acid	144.067	102, 87, 71	0.99	
14	3.556	C_5_H_6_N_5_O^+^	Guanine	Purine	152.0576	135, 110, 94	−1.25	[30]
15	3.647	C_19_H_23_O_6_^+^	Trihydroxy-dimethoxy-dimethylflavan	Flavonoid	347.1489	332, 329, 284, 227, 209	−0.04	
16	3.693	C_10_H_12_N_5_O_5_^−^	Guanosine	Purine	282.0862	150, 133	6.4	[30]
17	4.195	C_7_H_7_O^+^	Benzaldehyde	Aldehyde	107.0492	79, 77	3.12	
18	5.064	C_10_H_13_N_2_O_5_^−^	Thymidine	Pyrimidine	241.0838	208, 180, 150, 131	1.01	[30]
19	5.338	C_16_H_19_O_3_^+^	Methylbatatasin III	Phenolic	259.1326	234, 201, 160, 136	−1.04	[31]
20	5.338	C_10_H_11_N_4_O_6_^−^	Xanthosine	Purine	283.0712	197, 151, 66	9.86	[32]
21	5.566	C_19_H_23_O_6_^+^	Trihydroxy-dimethoxy-dimethylflavan isomer	Flavonoid	347.1521	332, 330, 318, 287, 251, 168	9.17	
22	6.343	C_8_H_12_N^+^	Phenylethylamine	Nitrogenous compound	122.0965	105	1.45	
23	6.846	C_15_H_17_O_9_^−^	*O*-caffeoylhexose	Phenolic acid	341.0912	179, 161	−3.31	[33]
24	6.891	C_12_H_21_O_4_^+^	Hydroxy-dihydrojasmonic acid	Organic acid	229.1443	170, 70	3.77	
25	8.034	C_10_H_13_N_2_O_4_^−^	Unknwon nitrogenous	Nitrogenous	225.0891	210, 181, 165, 139	−5.16	
26	8.125	C_10_H_12_N_5_O^−^	Zeatin	Purine	218.1048	204, 177, 146, 137	0.44	[34]
27	8.216	C_17_H_19_O_3_^+^	Hydroxy-methoxy-methylflavan	Flavonoid	271.1311	256, 228, 193, 177, 147, 137, 121	−6.53	
28	8.262	C_18_H_19_O_2_^+^	Phenylpropyl cinnamate	Ester	267.1374	189, 147, 120, 86	−2.08	
29	8.308	C_14_H_15_O^+^	Phenethylphenol	Phenolic	199.1099	135, 109	2.88	
30	8.354	C_9_H_11_O_3_^+^	Methoxyphenylacetic acid	Phenolic	167.0702	123, 109, 78	−0.42	[35]
31	8.765	C_13_H9O_9_^−^	Caffeoyl-*O*-dihydroxymaleic acid	Phenolic acid	309.0283	179, 135, 133	−9.93	
32	8.811	C_16_H_17_O_9_^−^	*O*-Caffeoylquinic acid	Phenolic acid	353.0911	191, 179	9.32	[15]
33	8.856	C_13_H_11_O_9_^−^	Caffeoyl tartaric acid	Phenolic acid	311.0438	179, 149, 135	−9.73	[36]
34	8.902	C_7_H_5_O_5_^−^	Gallic acid	Phenolic acid	169.0161	151, 125, 107, 95	−9.24	[37]
35	8.993	C_9_H_8_NO^+^	Unknwon	Aldehyde	146.0608	118, 91, 77	−3.73	
36	9.176	C_20_H_19_O_2_^+^	Unknown	-	291.1363	205, 190, 141, 128	−0.26	
37	9.359	C_13_H_11_O_8_^−^	*p*-coumaroyltartaric acid	Phenolic acid	295.0488	251, 163, 119, 87	9.69	[38]
38	9.404	C_10_H_10_N^+^	Unknown	Nitrogenous compound	144.0811	127, 116, 103, 91	−2.61	
39	9.633	C_14_H_13_O_9_^−^	Feruloyl-*O*-tartaric acid	Phenolic acid	325.0599	193, 134	−9.13	[39]
40	9.953	C_12_H_17_O_4_^−^	Hydroxyjasmonic acid	Organic acid	225.1157	165, 137, 97,81	−9.87	
41	9.999	C_9_H_5_O_4_^−^	Caffeic acid quinone	Phenolic acid	177.0209	162, 149, 135,121	−8.28	
42	10.09	C_12_H_12_NO_5_^−^	*p*-Coumaroyl-serine	Phenolic acid	250.0745	207, 161, 132, 115	−9.12	
43	10.227	C_8_H_7_N_4_O^−^	Unknown	Nitrogenous compound	175.0631	133, 105, 89, 77	−3.26	
44	10.273	C_9_H_7_O_4_^−^	Caffeic acid	Phenolic acid	179.0366	135, 124, 107, 93	5.95	[40]
45	10.501	C_11_H_12_NO_3_^−^	*N*-Acetyl-l-phenylalanine	Amino acid	206.0835	164, 147, 103, 91	−6	[41]
46	10.867	C_10_H_17_N_2_O_3_S^−^	Prolyl-methionine	Amino acid	245.0961	203, 186, 159, 142	0.27	
47	10.913	C_24_H_35_N_8_OS_4_^−^	Unknown	-	579.1791	254, 203, 116,72	0.75	
48	11.232	C_25_H_23_O_12_^−^	Scopoletin-7-*O*-dihexoside	Coumarin	515.1237	353, 191, 179, 173	8.15	[42]
49	11.552	C_9_H_15_O_4_^−^	Nonanedioic acid	Organic acid	187.0995	170, 125, 97, 87	7.29	[43]
50	11.849	C_10_H_11_O_4_^+^	Methyl caffeate	Phenolic acid	195.0657	177, 163, 150, 145	−2.98	[44]
51	12.009	C_20_H_31_O_6_^+^	Unknown	-	367.2151	349, 325, 204, 112	9.76	
52	12.329	C_15_H_11_O_6_^+^	Kaempferol	Flavonoid	287.0567	153, 135, 93	5.87	[45]
53	12.512	C_11_ H_17_O_3_^+^	Hydroxybutyl-methoxy-phenol	Phenolic	197.1188	180, 166, 153, 141	−9.75	
54	12.74	C_11_H_11_O_4_^−^	Unknown	Phenolic acid	207.068	179,161,135	8.29	
55	12.786	C_13_H_23_O_2_^+^	Tridecadienoic acid	Fatty acid	211.1694	151, 135, 83, 67	−0.79	
56	13.06	C_15_H_11_O_5_^+^	Apigenin	Flavonoid	271.0627	153, 131, 95	9.59	[46]
57	13.106	C_18_H_35_O_5_^+^	Trihydroxy-octadecenoic acid	Fatty acid	331.2476	288, 244, 175, 69	−0.9	[47]
58	13.151	C_18_H_33_O_5_^−^	Trihydroxyoctadecenoic acid isomer	Fatty acid	329.2364	311, 201,171	−9.19	[47]
59	13.288	C_13_H_21_O_2_^+^	Tridecatrienoic acid	Fatty acid	209.1542	163, 93, 79,71	−3.85	
60	13.334	C_15_H_23_O_2_^+^	Unknown terpene	Terpene	235.1712	219, 161, 121, 93	−5.11	
61	13.38	C_15_H_23_O^+^	Unknown terpene	Terpene	219.175	204, 175, 121, 79	−1.84	
62	13.562	C_20_H_33_O_5_^+^	Trihydroxyicosa-tetraenoic acid (lipoxin)	Fatty acid	353.2317	308, 277, 222, 199	−1.55	
63	13.7	C_18_H_29_O_4_^+^	Hydroxy-oxo-octadecatrienoic acid	Fatty acid	309.207	292, 273, 219, 165	1.31	[48]
64	13.837	C_18_H_27_O_3_^+^	Unknown	Terpene	291.1942	204, 177, 133, 119	5.55	
65	13.882	C_16_H_29_O_2_^+^	Hexadecadienoic acid	Fatty acid	253.2169	237, 209, 193, 174	−1.34	[49]
66	14.157	C_20_H_31_O_5_^+^	Dihydroxy-oxoicosa-tetraenoic acid	Fatty acid	351.2159	334, 149, 106, 81	−1.99	
67	14.385	C_45_H_83_O_16_P_2_^−^	Phosphatidylinositol phosphate (18:0/18:2)	Acyl glycerol	941.5213	880, 471, 394, 259	5.43	
68	14.431	C_15_H_13_O_3+_	Unknown	Terpene	241.0856	227, 211, 180, 157	−1.33	
69	14.705	C_18_H_27_O_2_^+^	Unknown	Terpene	275.2024	171, 147, 119,79	−6.62	
70	14.751	C_20_H_29_O_4_^+^	Unknown	-	333.2056	307, 188, 135, 87	−1.3	
71	14.842	C_21_H_23_O_8_^+^	Hexamethoxyflavone	Flavonoid	403.1394	388, 373, 212, 183	1.62	[50]
72	14.933	C_17_H_27_O_5_^−^	Glycerol-hydroxy-tetradecatrienoate	Acyl glycerol	311.1895	267, 179, 135	9.96	
73	14.979	C_18_H_27_O_3_^+^	Oxo-octadecatetraenoic acid	Fatty acid	291.1965	209, 157, 133, 121	−0.64	
74	15.208	C_18_H_31_O_4_^−^	Dihydroxy-octadecadienoic acid	Fatty acid	311.2245	267, 179, 135, 87	−6.15	[47]
75	15.436	C_20_H_21_O_7_^+^	Pentamethoxyflavone	Flavonoid	373.1281	358, 343, 312, 266, 197	−0.21	[50]
76	15.619	C_18_H_40_NO_3_^+^	Amino-octadecanetriol	Sphingolipid	318.2982	303, 242, 150, 62	−6.5	
77	15.847	C_13_H_23_O_3_^+^	Methyl dihydrojasmonate	Oxylipid	227.1645	212, 183, 141, 125	1.44	
78	15.893	C_20_H_33_O_4_^+^	Unknown	Terpene	337.2368	293, 232, 195, 168	−1.58	
79	15.938	C_18_H_29_O_3_^+^	Oxophytodienoic acid	Oxylipid	293.2117	238, 151, 145, 101	−0.02	
80	15.984	C_19_H_27_O_4_^+^	Unknown	Terpene	319.1893	303, 235, 165, 105	−3.4	
81	16.304	C_20_H_31_O_4_^+^	Unknown	-	335.2218	318, 273,158,83	0.34	
82	16.578	C_18_H_29_O_3_^−^	Hydroxylinolenic acid	Fatty acid	293.215	275, 249, 233, 183	−9.51	
83	16.578	C_18_H_29_O_2_^+^	Unknown	Terpene	277.2164	149, 121, 107, 93	0.85	
84	16.67	C_18_H_27_O_2_^+^	Unknown	Terpene	275.2015	149, 135, 121, 93	−2.45	
85	16.761	C_20_H_29_O_3_^+^	Unknown	Terpene	317.2088	299, 187, 164, 110	−7.31	
86	16.898	C_18_H_29_O_3_^+^	Oxooctadeca-trienoic acid	Fatty acid	293.2126	275, 248, 125, 93	−5.77	
87	17.081	C_13_H_25_O_3_^+^	Hydroxy-tridecenoic acid	Fatty acid	229.181	155, 109, 67	0.45	
88	17.172	C_18_H_37_O_6_^+^	Tetrahydroxy-octadecanoic acid (saturated)	Fatty acid	349.2602	306, 277, 190, 83	4.96	[51]
89	17.264	C_18_H_31_O_2_^+^	Octadeca-trienoic acid (Linolenic acid)	Fatty acid	279.2332	95, 81, 67	−7.17	
90	17.538	C_22_H_35_O_3_^+^	Unknown	Terpene	347.2564	276, 263, 174, 163	2.03	
91	17.583	C_18_H_29_O_2_^+^	Unknown	Terpene	277.2172	150, 121, 95, 81	−1.14	
92	17.629	C_22_H_37_O_3_^+^	Unknown	Terpene	349.2737	315, 275, 214, 165	−0.06	
93	17.995	C_24_H_41_O_5_^+^	Unknown	-	409.294	391, 353, 123, 86	−2.07	
94	18.132	C_18_H_21_O_4_^+^	Unknown	Terpene	301.1426	214, 182, 135, 93	−2.77	
95	18.269	C_18_H_31_O_3_^−^	Hydroxyolinoleic acid	Fatty acid	295.2303	277, 233, 195, 183	−8.22	
96	18.863	C_25_H_48_O_12_P^−^	Phosphoinositol (16:0/0:0)	Acyl glycerol	571.2935	434, 409, 380, 315	8.07	
97	19	C_24_H_39_O_4_^+^	Unknown	Terpene	391.283	373, 187, 85	−3.28	
98	19.183	C_19_H_33_O_2_^+^	Methyl-octadeca-trienoate	Fatty acid	293.2487	248, 169, 95	−3.5	[52]
99	19.228	C_22_H_35_O_2_^+^	Docosapentaenoic acid	Fatty acid	331.266	275, 235, 146, 67	8.58	
100	19.594	C_28_H_47_O_4_^+^	Unknown	Sterol	447.3459	429, 359, 206, 149	−2.2	
101	20.096	C_24_H_37_O_3_^+^	Unknown	Oxylipid	373.2719	356, 329,284,235	−4.8	
102	20.234	C_43_H79NO_10_P^−^	Phosphoserine (19:0/18:2)	Acyl glycerol	800.5521	741, 227	9.23	
103	20.371	C_16_H_31_O_3_^−^	Hydroxypalmitate	Fatty acid	271.2302	227, 225, 195, 151	−7.7	
104	20.417	C_43_H_76_O_12_P^−^	Phosphoinositol (P-16:0/18:4)	Acyl glycerol	815.5059	758, 475, 428, 281	−2.56	
105	20.599	C_45_H_81_NO_10_P^−^	Phosphoserine (19:1/20:2)	Acyl glycerol	826.566	767, 477,279	6.82	
106	20.691	C_34_H_68_NO_5_^−^	Ceramide (t18:0/16:0(2OH))	Sphingolipid	570.5157	314, 255	9.46	[53]
107	21.056	C_30_H_49_O_2_^+^	Unknown	Sterol	441.369	423, 361, 259,219	−8.39	
108	22.472	C_42_H_84_NO_5_^+^	Ceramide (t18:1/24:0(2OH))	Sphingolipid	682.6342	665, 427, 372, 162, 74	−0.29	[53]
109	22.747	C_48_H_92_NO_10_^−^	Iotroridoside	Glycosphingolipid	842.6782	438, 179, 89	6.56	[54]
110	22.838	C_43_H_77_O_13_P^−^	Phosphatidylinositol (16:0/18:2)	Acyl glycerol	832.5031	520, 366, 273	−9.16	
111	22.93	C_43_H_74_O_13_P^−^	Phosphatidylinositol (14:0/20:4)	Acyl glycerol	829.4852	787, 225, 89	−2.47	
112	23.295	C_45_H_74_O_13_P^−^	Phosphatidylinositol (16:1/20:5)	Acyl glycerol	853.4843	610, 339, 108	−3.45	
113	23.706	C_43_H_76_O_13_P^−^	Phosphatidylinositol (20:1/14:1)	Acyl glycerol	831.5004	772, 459,80	−3.01	

## Supplementary Materials

The following are available online. Table S1: Toxicological study of different doses of *L. nudicaulis* ethanolic extract administered orally in mice.

## Sample Availability

Samples of the compounds are not available from the authors.
