# Supplementary material for: Chemical Profile of Launaea nudicaulis Ethanolic Extract and Its Antidiabetic Effect in Streptozotocin-Induced Rats
Source: Molecules. 2021 Feb 13;26(4):1000. doi: 10.3390/molecules26041000 (PMC7918448; doi:10.3390/molecules26041000)
Supplement: Supplementary file 1 [file molecules-26-01000-s001.pdf]

Supplemntray Materiels

Table S1. Toxicological study of different doses of *L. nudicaulis* ethanolic extract administered orally in mice.

Groups	Dose/Day	Mortality during 48 h
Group I	Saline (10 mL/kg)	0/8
Group II	1 g/kg	0/8
Group III	2 g/kg	0/8
Group IV	3 g/kg	0/8
Group V	4 g/kg	1/8
Group VI	5 g/kg	4/8
Group VII	6 g/kg	6/8
